# Genotyping-by-Sequencing in Plants

**DOI:** 10.3390/biology1030460

**Published:** 2012-09-25

**Authors:** Stéphane Deschamps, Victor Llaca, Gregory D. May

**Affiliations:** 1 DuPont Agricultural Biotechnology, Experimental Station, PO Box 80353, 200 Powder Mill Road, Wilmington, DE 19880-0353, USA; Email: victor.llaca@usa.dupont.com; 2 DuPont Pioneer, 7300 NW 62nd Ave., P.O. Box 1004, Johnston, IA 50131-1004, USA; Email: gregory.may@pioneer.com

**Keywords:** genotyping, genotyping-by-sequencing, sequencing, next-generation sequencing, genome-wide association, DNA, SNPs, NGS, GBS

## Abstract

The advent of next-generation DNA sequencing (NGS) technologies has led to the development of rapid genome-wide Single Nucleotide Polymorphism (SNP) detection applications in various plant species. Recent improvements in sequencing throughput combined with an overall decrease in costs per gigabase of sequence is allowing NGS to be applied to not only the evaluation of small subsets of parental inbred lines, but also the mapping and characterization of traits of interest in much larger populations. Such an approach, where sequences are used simultaneously to detect and score SNPs, therefore bypassing the entire marker assay development stage, is known as genotyping-by-sequencing (GBS). This review will summarize the current state of GBS in plants and the promises it holds as a genome-wide genotyping application.

## 1. Introduction

The analysis of genomic variation is an essential part of plant genetics and crop improvement programs. DNA polymorphisms can be directly related to phenotype differences, be genetically linked to its causative factor, or indicate relationships between individuals in populations [1]. Over the last 30 years, the use of genotyping has enabled the characterization and mapping of genes and metabolic pathways in plants as well as the study of species diversity and evolution, marker-assisted selection (MAS), germplasm characterization and seed purity. Single Nucleotide Polymorphisms (SNPs) have emerged as the most widely used genotyping markers due to their abundance in the genome and the relative ease in determining their frequency in a cost-effective and parallel manner in a given panel of individuals. 

The field of agricultural genomics is in the midst of a technological revolution caused by the relatively sudden emergence of “next-generation” DNA sequencing technologies, driven in part by the completion of the human genome and the desire to apply the benefits of genomics to a better understanding of diseases and a more personalized view of medicine. By greatly reducing limitations in generating sequence information, these technological advances have facilitated the characterization of genes and genomes, and started to provide a more comprehensive view of diversity and gene function in plants. The increased ability to sequence in a cost-effective manner large numbers of individuals within the same species has altered the concept of variant discovery and genotyping in mapping studies, especially in plant species with complex genomes or limited public resources available. A new concept, namely genotyping-by-sequencing (GBS), has emerged, where the detection of sequence differences (namely SNPs) in a large segregating or mutant population is combined with scoring, thus allowing a rapid and direct study of its diversity targeted towards the mapping of a trait or a mutation of interest. This review will summarize the current state of genotyping and next-generation DNA sequencing technologies then provide some examples of studies where next-generation DNA sequencing has been used in plant species for genotyping applications.

## 2. Genotyping Applications

The first plant DNA markers were based on restriction fragment length polymorphisms (RFLPs) [2]. Early hybridization-based, radioactive RFLP techniques were inherently challenging and time consuming, and were eventually replaced by less complex, more cost-effective PCR-based markers. Among them, simple-sequence repeats (SSR) [3] were particularly useful as genetic markers. They were relatively inexpensive, abundant in plant genomes and more informative than bi-allelic markers. Additional marker strategies were developed using different combinations of PCR, restriction digestion and gel electrophoresis techniques. Major marker techniques included random amplification of polymorphic DNAs (RAPDs) [4]; sequence characterized amplified region (SCARs) [5]; cleaved amplified polymorphic sequences (CAPS) [6]; Intersimple Sequence Repeats (ISSRs) [7]; amplified fragment length polymorphisms (AFLPs) [8]; and direct amplification of length polymorphism (DALP) [9]. The improvement of Sanger sequencing throughput in the 1990’s, in combination with the start of genome and expressed sequence tag (EST) sequencing programs in model plant species, led to the acceleration in the identification of variation at the single base pair resolution [10]. The use of single-nucleotide polymorphisms (SNPs) as markers for genotyping using direct re-sequencing increased the potential to score variation in specific targets. More importantly, the increase in information about potentially millions of genome-wide SNPs or small insertion-deletions and their surrounding sequence context set the foundation of high-throughput genotyping. 

For the past 15 years, automation and miniaturization in SNP-based marker technologies has increased marker density and reduced genotyping costs and time by orders of magnitude in relation to earlier approaches. Some of the most commonly used systems are based on fluorescent detection of SNP-specific hybridization probes on PCR products such as Taqman, Molecular Beacons and Invader [11,12,13,14,15,16]. Other strategies such as Sequenom homogeneous Mass Extend (hME) and iPLEX genotyping systems involve MALDI-TOF mass spectrophotometry of SNP-specific PCR primer extension products. These technologies originally allowed data collection of hundreds to up to a few thousand SNP/samples per day/machine. With the increasing need for higher throughput, end-point fluorescent assays such as Taqman and Invader have been significantly enhanced by the use of array tape technology in place of 96, 384 or 1,536-well microtiter plates, reducing cost per assay to a fraction of the original reaction and increasing throughput in a format that allows the PCR processing of the equivalent of hundreds of microtiter plates on one roll of tape [17]. 

The emergence of massively parallel array systems has further enabled parallel scoring of up to hundreds of thousands of markers in plants [18]. These ultra-high throughput technologies are wide-ranging and researchers can now select methods based on application, assay simplicity, cost, throughput and accuracy. Among the most widely used array-based systems in plants are the GoldenGate and Infinium assays, which consist on multistep protocols based on Illumina’s BeadArray/BeadChip technology. The GoldenGate system allows screening of a large number of samples using a single multiplexed assay that can include as many as 3,072 SNPs. The Illumina's Infinium provides considerably higher throughput, of up to four million SNPs on a single sample, or up to several hundred thousand on multiple samples in the same array. In Infinium, samples are incubated on bead chips where they anneal to locus-specific 50-mers covalently linked to beads. After hybridization, oligos are subject to allele-specific single-base extension; followed by fluorescent staining, signal amplification, scanning in a dual-color channel reader, and analysis. A major advantage of Infinium is the availability of commercially available validated chips in selected species, such as the MaizeSNP50, which includes more than 56,000 SNP markers derived from the comparison of the B73 maize reference genome sequence to multiple lines. The use of pre-made arrays reduces cost considerably although the actual number of markers derived from this array will be considerably lower, depending on the relationship to the reference and gene representation in the interrogated plants. Other arrays provide comparable levels of throughput. Beckman Coulter's GenomeLAb SNPstream allows the processing of up to three million genotypes in 384 samples per day per instrument. The widely used Affimetrix GeneChip system cannot only detect hundreds of thousands of SNPs in a single array but it can also be used for SNP discovery by sequencing by hybridization (SbH). 

## 3. Ultra-high Throughput Genotyping Applications

In plant genetics, not all marker-related applications require massively parallel, genome-wide genotyping. Plant phylogenetic and diversity studies have successfully exploited relatively low marker densities or regional markers to determine relationship in plants at the interspecific and intraspecific levels [19,20]. The ability of highly polymorphic SSRs and AFLPs to differentiate individuals in a population has made them markers of choice for pedigree analyses and cultivar identification. In linkage mapping, relatively low marker density has been sufficient to enable the mapping and characterization of simple traits and quantitative trait loci (QTL) with large effects in the total genetic variance. Recent linkage mapping studies in maize have identified QTL with relatively large effects in oil content [21] and root architecture [22]. However, in most cases QTL characterization by linkage mapping can be problematic as intervals may encompass large genetic and physical distances and require walking through several megabase-pairs of sequence, with a large number of potential candidates [23,24,25]. Only a small fraction of mapped plant QTL has been cloned by linkage mapping due to the low resolution of available mapping strategies [26,27]. Increasing marker density may not provide additional benefits as map resolution can be limited by the relatively few recombinants generated from two original parents in a limited number of generations and progeny as polymorphisms are identified between two parents and then followed in a segregating population. Finally, medium to high marker densities may be required in marker assisted selection (MAS) to allow early testing of specific traits using linked markers to reduce breeding time and number of plants and space needed. In cases of selection for specific traits to reduce linkage drag or pyramiding genes for the same trait, the use of low density or regional markers may be sufficient. 

The development of ultra-high-throughput genotyping technologies and later the emergence of new sequencing platforms has enabled the development of high-density applications in QTL characterization and plant breeding that had been difficult to accomplish or not feasible before. Major marker-intensive applications include genome-wide association studies (GWAS) and bulked segregant analysis (BSA) [28,29]. Unlike linkage mapping, GWAS exploits the natural diversity generated by multi-generational recombination events in a population or panel [30,31,32,33]. This strategy can result in increased resolution compared to linkage mapping populations, as long as enough markers are provided. Only the markers that are in linkage disequilibrium (LD) with the trait of interest will show association to such trait. Without enough genome-wide marker coverage, association mapping studies need to focus on polymorphisms in candidate genes that are suspected to have roles in controlling phenotypic variation for one specific trait of interest [34]. The concept of GWAS predates high throughput genotyping technologies and the first genome-wide association study in plants was conducted more than 10 years ago in sea beet (*Beta vulgaris* ssp. *maritima*) [35]. However, the effective implementation of genome-wide association mapping in plants and the determination of optimal marker density have been problematic because of lack of knowledge regarding the degree and structure of LD distribution in specific target populations. LD can be affected by multiple factors at the species or population level, including the degree of selfing, epistasis, admixture and population bottlenecks followed by genetic drift. Complex breeding history and limited gene flow are common factors in plants generating stratification and uneven distribution of alleles in populations, which can lead to false associations [36,37,38]. Depending on the size and LD characteristics of the population under analysis, tens of thousands or even millions of independent genetic markers may be needed to correct the effects of population structure and achieve optimal resolution in a genome-wide scan.In plant populations with low LD, genotyping costs have been a serious limiting factor thus far, deeming candidate-gene association analysis the method of choice. In plants, the ability to create lines of individuals with identical or near identical background offer the potential to create public GWAS resources that can be accessed by multiple groups and rapidly resolve complex traits. Plant GWAS can be performed in large numbers of samples in replicated trials using inbreds, double haploid (DH) lines and recombinant inbred lines (RILs) [39]. The determination of large numbers of genome variants in combination with transcription profiling can be used to determine expression quantitative loci (eQTLs) [40], mapping regions with cis- and trans-effects [41,42,43,44]. Bulked segregant analysis can be used as a time- and cost-effective way to identify markers associated to specific phenotypes without the need of having a linkage map or sampling large numbers of samples in a population [45]. BSA can be used for extreme mapping, where plants from extreme ends of the phenotype range in a population derived from a single cross are bulked, or pooled, and the genotype differences correlated to the trait of interest. This method allows the easy detection of QTL in large populations as long as the number of markers available is large and widely distributed along the genome [46].

Finally, the increase in availability and cost reduction of markers has made feasible the concept of Genomic Selection for plant breeding. Genomic, or Genome-wide, Selection (GS) [47] has been proposed as an effective method to breed for traits involving multiple QTL with low heritability. In GS, unlike GWAS, arrays of markers are selected without establishing association with traits, and are used to predict phenotypes. Only genotypic data need to be used in a breeding population as predictor to select individuals with the best breeding values. In GS, an initial training population is used to capture both phenotypic and genotyping data from a very large number of markers, to capture all additive genetic variance for specific traits. Breeding values are then estimated in a breeding population solely based on genotype and the estimated marker effects.

With all their potential to increase SNP density and resolution in large samples for GWAS, BSA and GS, current array-based technologies have clear limitations. They require prior generation of sequence information, identification of polymorphisms, validation and array production. The value of Sanger or NGS-driven massive polymorphisms discovery and ultra-high throughput platforms can be seriously restricted by cost and time limitations in the design, validation and deployment of molecular markers. Furthermore, the significant sequence diversity and the high structural polymorphism observed in important plant models such as maize imposes a challenge to these knowledge-based platforms. Structural genome differences, including translocations, copy number variation and presence-absence variation are observed in landraces and lines in the same species and correspond to differences in repetitive, non-coding DNA and gene content [48,49]. There is an inherent bias towards cultivars used as reference in genome projects. Molecular markers may be absent near or within the gene space located in larger structural variations (*i.e.*, CNVs, PAVs, and large indels). With the falling cost of NGS there is an increased interest in genotyping-by-sequencing (GBS), where obtained sequence differences are used directly as markers for analysis. We will describe here a number of GBS strategies applied to populations or panels in plants genetics and breeding.

## 4. Ultra High Throughput DNA Sequencing

The field of DNA sequencing recently has been marked by dramatic increases in throughput combined with a significant decrease in cost per base of raw sequence. For over two decades, the advent of the modern genomics era has been characterized by major prokaryotic and eukaryotic genome sequencing projects achieved using the Sanger method of sequencing [50,51]. Sanger sequencing still is the gold standard in terms of generating high quality sequencing information as many finished-grade whole genome sequencing drafts were achieved using that method [52,53,54,55,56,57,58,59]. Sanger sequencing also can be used to discover genetic variations (including SNPs) within a set of individuals in a population. One particular method employs the PCR amplification of genomic DNA in multiple individuals as a mean to generate homologous DNA fragments that are end-sequenced and compared to reveal particular sequence variations [60,61]. Since its inception more than 35 years ago, the Sanger sequencing method has gone through several iterations of improvements, including automated sequencers [62,63] and the emergence of fluorescent dye terminators to capture nucleotide incorporation events [64,65]. However, the high costs and labor generally associated with the Sanger sequencing technique fundamentally limit its reach and use in large multi-genome comprehensive studies, both for medical and agricultural applications. These limitations have contributed to the emergence in the past eight years of “next-generation sequencing” (NGS) technologies that rely on massively parallel sequencing and imaging techniques to yield several hundreds of millions to several hundreds of billions of bases per run [66]. Current NGS platforms can be divided into two categories, labeled as “second-generation” and “third-generation”, depending, mostly, on whether DNA templates are amplified on an immobilized support prior to sequencing, and the subsequent generation of sequencing data either from clustered copies originating from the same DNA strand, or directly from single DNA molecules [67].

Second-generation DNA sequencing strategies (Table 1) all follow a similar pattern for DNA template preparation, where universal adapters are ligated at both ends of randomly sheared DNA fragments. They all also rely on the cyclic interrogation of millions of clonally amplified DNA molecules immobilized on a synthetic surface to generate up to several billions of sequences in a massively parallel fashion. Sequencing is performed in an iterative manner, where the incorporation of one or more nucleotides is followed by the emission of a signal and its detection by the sequencer [68]. 

**Table 1:** Comparison of representative next-generation sequencing technologies.

Sequencing Platform	Sequencing Chemistry	Detection Chemistry	Run Time^a^	Read Length (bp)	Reads per Run (million)	Throughput per Run (Gbp)
Roche 454 FLX Titanium	Sequencing by Synthesis	Light	23 hours	~800	~1	~0.7
Illumina MiSeq	Sequencing by Synthesis	Fluorescence	39 hours	2 × 250^ b^	~1	~8
Illumina HiSeq2500	Sequencing by Synthesis	Fluorescence	11 days (high output)/27 hours (rapid run)	2 × 100^ b^	~3,000	~600 (high output)/~120 (rapid run)
Life Technologies 5500xl	Sequencing byLigation	Fluorescence	8 days	75 + 35 ^b^	~5,000	~310
Ion Torrent PGM	Sequencing bySynthesis	pH	4 hours	100	1	~0.1

^a ^Not including library construction; ^b^ Paired-end read sequencing.

The first NGS platform to become commercially available was the Roche 454 GS20 sequencer, which later was replaced by the 454 GS FLX Titanium sequencer [69].The 454 technology combines clonal amplification of a single DNA molecule by PCR in a water-oil emulsion [70] with a sequencing-by-synthesis approach known as pyrosequencing [71].Here, the sequential release of single nucleotides, followed by the detection of pyrophosphate release during nucleotide incorporation, generates series of chemiluminescent signals whose intensities are used to determine the number of bases being incorporated to the elongating DNA strand. The 454 FLX Titanium sequencer currently is capable of generating approximately 450Mbp of sequences per 10-hour run with a read length up to 600bp and 99.99% accuracy [72]. The NGS technology commercialized by Illumina [73] generates shorter reads, ranging from 50 to 150bp, with sequencing throughputs ranging from ~1.5Gbp to ~600Gbp depending on the platform being used. Several instruments are commercialized by Illumina, ranging from the bench top MiSeq sequencer to the high-throughput HiSeq2500 sequencer. The Illumina sequencing technology combines clonal amplification of a single DNA molecule with a cyclical sequencing-by-synthesis approach. The PCR amplification is performed using a solid phase amplification protocol, also known as “bridge amplification” [74], to generate up to 1,000 copies of an original molecule of DNA, grouped together into a cluster. Sequencing is performed with proprietary reversible fluorescent terminator deoxyribonucleotides, in a series of cycles consisting of single base extension, fluorescence detection (where the nature of the signal is used to determine the identity of the base being incorporated) and cleavage of both the fluorescent label and of chemical moieties at the 3’ hydroxyl position to allow for the next cycle to occur. Another NGS manufacturer, Life Technologies, currently offers two series of NGS instruments: the large-scale 5500 series, whose yields and read lengths are up to >20Gbp per day and 75bp, respectively [72], and the small-scale Ion Torrent series, yielding up to 10Gbp per run in less than a day [75]. The Ion Torrent series of instruments (PGM and Ion Proton) are smaller instruments that use semi-conductor chip technology to capture a signal after incorporation of a single base to the elongating strand of DNA. Similarly to the 454 sequencing technology, DNA fragments flanked by universal adapters are clonally amplified by emulsion PCR prior to being sequenced, and sequencing is performed by releasing single nucleotide types sequentially and detecting the release of one or more protons, and the subsequent local change in pH, during nucleotide incorporation. 

Applications of second-generation sequencing technologies are numerous and include *de novo* assemblies of prokaryotic and eukaryotics genomes [76,77], alignment and comparison or targeted regions for variant discovery [78,79,80,81], profiling of transcripts [82,83,84] and small RNAs [85,86,87], profiling of epigenetics patterns [88,89] and chromatin structure [90,91], and species classification via metagenomics studies [92]. 

## 5. Applications of NGS Technologies to Genotyping-by-Sequencing

**Figure 1:**
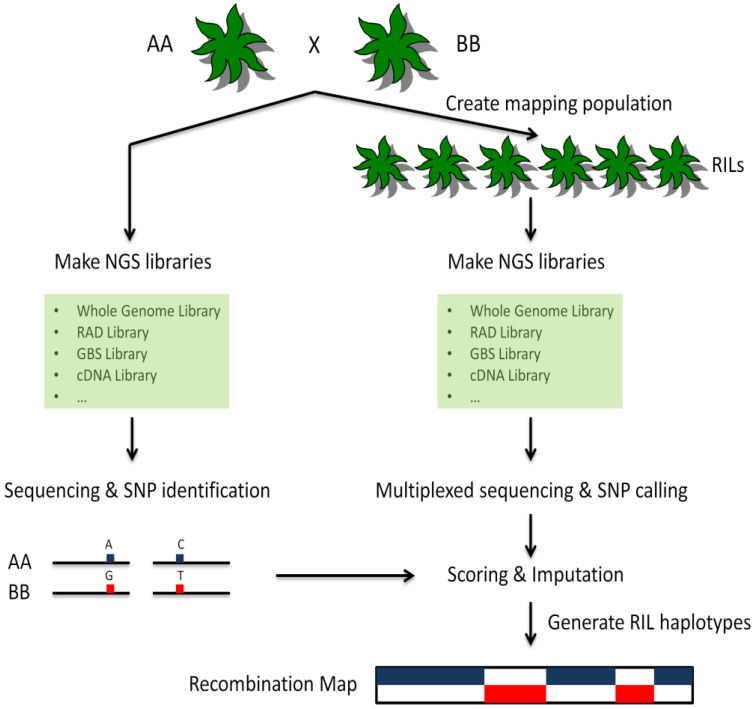
Schematic diagram of a representative GBS procedure. Two parents (AA and BB) are selected to create a mapping population. The parents are deeply sequenced using NGS technologies. SNPs and other variations between them are identified. The RILs are prepared using the same library construction strategy as the two parents (see text for details) and sequenced at lower coverage using NGS technologies. The resulting sequences are used to determine allelic diversity for each individual. Genotypes are assigned based on parental information. Haplotypes and recombination maps are created for each RIL. Blocks of haplotypes can be used directly as markers for mapping applications.

As described in a previous paragraph, the development of markers, as well as their scoring across populations, traditionally has been a high-cost process with many labor-intensive and time-consuming steps. The emergence of SNP arrays has reduced the time and efforts spent on scoring but the development of new markers still requires significant investments. These markers also are specific to the population in which they are developed, and the resulting allelic bias can be problematic in some divergent populations and species. Preliminary sequence information of regions flanking a SNP of interest also is required to develop marker assays, and only a few SNPs derived from sequencing data generally can be considered suitable for marker development, due to several factors, including proximity to repetitive regions, to known markers or to other regions of interest. By contrast, as chemistry and software improvements are leading to significant decreases in the overall cost of NGS, resequencing extended to entire populations, rather than to a few parental individuals for the sole purpose of discovering variants, enables the simultaneous genome-wide detection and scoring of hundreds of thousands of markers [93]. This “genotyping-by-sequencing” (GBS) approach also uses data directly from the populations being genotyped, thus removing ascertainment bias towards a particular population. A typical GBS procedure is shown in Figure 1. Genetic maps generated using GBS-based sequencing information then can be used subsequently for identifying loci of interest from different sets of individuals, including segregating populations or mutant pools. 

GBS can be performed either through a reduced-representation or a whole-genome resequencing approach. The presence of repetitive elements in plants [94] can represent a significant challenge for *de novo* assembly, alignment to a reference sequence and sequence comparison for variant discovery. The choice of whether to sequence the entire genome or a reduced portion of it is generally dictated by several factors, including repetitive content, ploidy, and presence or absence of homeologs [95,96]. Whole genome resequencing has been performed in Arabidopsis [97] and rice [98]. In larger and more complex genomes, such as maize [93] or wheat [99], where much of the sequence is repetitive, the use of reduced-representation resequencing is generally preferred. Several strategies are available for reducing the complexity of a genome. The “mRNA-Seq” strategy, where cDNA molecules are chemically cleaved and the resulting fragments are end-sequenced, is an effective way of targeting coding regions of the genome [100]. Other genome reduction approaches are based on the distinct methylation pattern of plant genomes [101,102] and include the use of methylation-sensitive restriction endonucleases to enrich for low-copy hypomethylated regions of the genome [103,104]. Other strategies for genome reduction such as multiplexed amplification of target sequences [105], molecular inversion probes (MIPs) [106] or the use of probes to capture DNA fragments by direct hybridization prior to sequencing [107,108] are available but can be labor intensive and rely heavily on existing sequence information, thus potentially limiting their value in large and highly divergent populations or species.

## 6. Polymorphism Detection from NGS Data

SNP calling and genotyping of NGS data must take into accounts characteristics inherent to NGS technologies. NGS data typically have a higher error rate than traditional Sanger sequencing or SNP genotyping methods. This traditionally has been addressed via deeper sequencing, thus increasing the confidence that a particular SNP call is correct. However, recent GBS studies, where whole genomic samples from large mapping populations are sequenced, have mostly relied on low sequencing coverage [98]. Li *et al.* [109] also have developed models showing that sequencing many individuals at low depth (2–4×) was a powerful alternative strategy to sequencing few individuals at high depth (30×) for complex trait association studies (it must be noted that such a low coverage model assumes a diploid genome and therefore needs to be evaluated in plants, where genome duplication and polyploidy are prevalent). NGS technologies also generate shorter reads than Sanger sequencing, thus increasing the risk of aligning a particular sequence to the wrong region. For all these reasons, calling SNPs within a population remain challenging. Several algorithms have been developed to address these issues and improve confidence for SNP calls or genotypes in relation to a particular read aligned to a reference sequence. While some methods are simply based on filtering out low quality data and counting alleles for a given locus, others are more probabilistic in nature, incorporating errors introduced during basecalling, alignment and assembly and coupling them with existing data such as allele frequencies, reference haplotypes or linkage disequilibrium information [110]. Table 2 provides several examples of software that have been developed for SNP detection from NGS data.

**Table 2:** Non-exhaustive list of available SNP calling software for NGS data.

Software	Link	Input Format	Primary Function
MAQ	http://maq.sourceforge.net/	FASTA, FASTQ	Mapping and Assembly
SAMtools	http://samtools.sourceforge.net/	SAM, BAM	Alignments
GATK	http://www.broadinstitute.org/gatk/	SAM	Alignments
SOAPsnp	http://soap.genomics.org.cn/soapsnp.html	SOAP	Mapping and Assembly
SNIP-Seq	http://polymorphism.scripps.edu/~vbansal/software/SNIP-Seq/	Pileup	Alignments
MapNext	http://evolution.sysu.edu.cn/english/software/mapnext.htm	FASTA, FASTQ	Alignments

While SNP calls can be performed independently at each locus, linkage disequilibrium (LD) often can be used to impute missing data in SNP datasets for a particular population. LD is defined in population genetics as the non-random association of linked alleles at two or more loci, and typically exists within regions located on the same chromosome. As a consequence, entire haplotypes often can be defined by a few SNPs located at the boundaries of such regions in LD, and individuals within a recombinant population sharing alleles flanking a region of interest are expected to share the same haplotype within this region. As a consequence, several statistical methods have been developed for genotype imputation in panels of related individuals (using identity-by-descent information in linkage and association studies) and unrelated individuals (by comparing markers to a reference panel of haplotypes) (for review, see [111]). It must be noted that the use of imputation to estimate the genotype of many individuals that are not directly sequenced in specific areas is central to the concept of GBS, as biological and technical bias during sample preparation and sequencing generally lead to variable sequencing coverage at a particular locus between individuals. 

## 7. Genotyping-by-Sequencing in Plants

Many traits in plants, such as yield, are quantitative, resulting from the combinatorial effect of many genes [112]. The mapping of underlying quantitative trait loci (QTL) has been made possible by the emergence of molecular markers, genotyping technologies and related statistical methodologies [1]. Initially, the identification of QTL was mostly based on linkage mapping strategies, where polymorphisms between two parents are detected in a segregating population, and the linkage of a particular region to a given phenotype can be determined by genotyping recombinants exhibiting phenotypic variations for a trait of interest [21,113]. However, the relatively small number of recombinants generated from two parents in a limited number of generations means that linkage mapping generally has low resolution, encompassing very large genetic and physical distance, with many possible candidate genes for a trait of interest. This has led to the emergence of association mapping studies, which utilize the natural diversity present in a multi-generational population and provides higher resolution than linkage mapping populations to map traits of interest [28,114,115]. Larger genome-wide association studies (GWAS) require hundreds of thousands to millions of markers to generate sufficient information and coverage, and getting such resolution has been greatly enhanced by the emergence of NGS technologies [116,117,118]. More recently, NGS technologies have been used to resequence collections of recombinant inbred lines (RILs) to analyze, in correlation to appropriate phenotypic values, and map various traits of interest in specific environments. Such resources have been generated in maize [31,119] where a collection of 5,000 RILs derived from a nested association mapping (NAM) population have been resequenced using a restriction endonuclease-based reduced-representation approach and the Illumina sequencing technology, generating a total of 1.4 million SNPs and 200,000 indels. However, such resources have limited value beyond their population and a number of related sequencing protocols have been applied to other population for linkage mapping, association mapping, or bulked segregant analysis studies*.* In rice (*Oryza* sativa), Huang *et al.* [98] re-sequenced with the Illumina technology 150 F_11_ RILs derived from a cross between Indica and Japonica rice cultivars. The resulting sequences, generated after whole-genome re-sequencing of each RILs to an average 0.02X coverage, resulted in the discovery of 1,226,791 SNPs, separated by an average of 40Kbp. Haplotypes and recombination breakpoints could be determined for each RIL, using the parental origins of SNPs in discrete regions of the genomes, and a recombination bin map made of 2,334 bins for the 150 RILs was constructed from the haplotypes. Using each bin as a genetic marker, 49 QTLs linked to various phenotypes in rice could be detected, including 5 QTLs physically located at positions overlapping with the location of candidate genes described in previous studies [120]. 

Construction of a low-density GBS linkage map using the reduced-representation sequence-based marker discovery technique known as restriction site associated DNA sequencing (RAD) [121] has been reported in barley in barley (*Hordeum vulgare*) [122]. The RAD approach does not require any prior knowledge of the genome of the species being investigated and produces two types of markers: (1) co-dominant markers from sequence variations present in short targeted regions of the genomes immediately adjacent to selected restriction endonuclease cutting sites and (2) dominant markers from sequence variations present within the selected restriction endonuclease cutting sites. RAD sequencing was used to generate a set of 530 fixed SNP markers from the Oregon Wolfe Barley (OWB) parental inbred lines. These markers were classified as codominant, selected from approximately 10,000 clusters of RAD sequences obtained on the Illumina Genome Analyzer, and compared between lines using a k-mer algorithm allowing 0, 1 or 2 mismatches for every 28bp of sequence. After having excluded 94 markers from the analysis due to missing data and absence of linkage, the remaining 436 markers then were used to score RAD sequences obtained from a set of 93 individuals from a double haploid (DH) OWB mapping population and assist in the construction of a linkage map with an average marker density of 5cM. The RAD map and a higher density map generated by combining RAD markers with 2,383 markers previously reported by Szücs *et al.* [123] both allowed for the detection of the same large-effect QTLs for reproductive fitness traits, confirming the value of RAD markers for developing linkage maps and QTL mapping. RAD sequencing also was used in perennial ryegrass (*Lolium perenne*) to construct a linkage map and detect QTLs associated with resistance to stem rust caused by the pathogen *Puccinia graminis* subsp. *graminicola* [124]. The obligate outcrossing mating system of *Lolium*, resulting in high levels of heterozygosity and population heterogeneity, makes any attempt at marker development a challenging endeavor. A pseudo-testcross approach [125] combined with sequence-based marker development was tested for the identification of markers associated with stem rust resistance. RAD sequencing was performed on 188 F_1_ individuals, following the development of 1,733 RAD markers, characterized by 1 or 2 bi-allelic SNPs or small indels, from the resistant (male) and susceptible (female) parental lines. The analysis of the F_1_ RAD data led to the identification of 329 RAD markers for the female map and 305 RAD markers for the male map, with an average distance between markers of 2.3cM and 2.6cM for the female and male maps, respectively. Three QTL for stem rust resistance were subsequently identified in this population from linkage maps generated from the selected RAD markers, combined with SSR and STS markers [126,127,128,129] and tested against parental DNA and a random panel of six F_1_ progeny DNA. Finally, the RAD approach was used in narrow-leafed lupine (*Lupinus angustifolius*) to discover new markers closely associated with a single dominant gene, known as *Lanr1*, conferring resistance to anthracnose caused by the pathogen *Colletotrichum lupini* [130]. RAD sequencing first was performed on 20 F_8_ individuals and the two resistant and susceptible parents of a mapping population. A total of 38 co-dominant RAD markers were selected as candidate markers linked to the *Lanr1* gene, from an initial pool of 8,207 putative SNP markers. Resistant and susceptible alleles for all 38 markers were confirmed from both the parental and progeny RAD marker data. A subset of 5 RAD markers then were converted into PCR-based markers exhibiting co-dominant polymorphic bands on SSCP gels and tested on 186 F_8_ individuals. Linkage analysis using the PCR-based marker genotyping score data and the anthracnose phenotyping data confirmed that all five newly developed markers were linked to the *Lanr1* gene, including two markers flanking the gene within 0.9cM, thus enabling a very simple and efficient assay for marker-assisted selection in lupine breeding programs.

Another important application derived from RAD markers has been the development of SSR markers for mapping purposes. Barchi *et al.* [131] generated RAD sequences in eggplant from a pair of mapping parents, enabling the discovery of ~10,000 SNPs, out of which a representative subset of 384 was used for fingerprinting a panel of eggplant germplasm using an Illumina GoldenGate assay. In the same study, RAD sequences also led to the identification of ~2,000 putative SSR markers that have been applied for genetic mapping and diversity analysis. Readers are directed towards a review article by Zalapa *et al.* [132] for more information on SSR marker identification in plants using NGS technologies.

Data shown above clearly confirm the value of reduced-representation sequencing approaches such as RAD sequencing for variant discovery and genotyping by sequencing, including in species with very limited public resources. Elshire *et al.* [93] demonstrated the feasibility of another reduced-representation highly-multiplexed GBS strategy in the complex genomes of maize (*Zea mays*) and barley (*Hordeum vulgare*) using a simple procedure targeting regions flanking restriction endonuclease sites. The approach included digestion of genomic DNA with a methylation-sensitive restriction endonuclease followed by ligation to barcoded adapters, pooling, PCR-based amplification, and sequencing of the amplified pool on a single lane of an Illumina flow cell. In maize, two parents and 276 RILs from the maize IBM (B73 × Mo17) mapping population were sequenced on six lanes of a single Illumina flow cell at 48-plex. A total of 809,651 sequences occurring at least five times and aligning uniquely to the reference genome were selected and generated a total of 25,185 bi-allelic SNPs that were added to a reference map. No alternate allele was found for 584,119 sequences and, by treating these as dominant data, an additional 167,494 markers were added to the map, out of which 133,129 uniquely aligned to the reference genome. In barley, two parents and 43 double haploid lines from the OWB mapping population were sequenced on one lane of an Illumina flow cell. A total of 2.1 million reads present in at least 20% of the RILs were selected and mapped to the OWB framework map by considering sequences as dominant markers. Prior to mapping, the genetic map was collapsed to retain 436 bi-allelic markers containing unique linkage information in the 43 lines. A total of 24,186 sequences then were mapped and, for 4,596 of them present in one of the lines, 99% agreed on parental origin with the reference markers. A modified version of the protocol described in Elshire *et al.* [93] was successfully applied to the complex genomes of barley and wheat [133]. SNP detection in wheat, as in barley, is a challenging endeavor for multiple reasons. First, the very large genome sizes (~16Gbp for hexaploid wheat *vs.* ~0.13Gbp for Arabidopsis) warrant using a reduced-representation strategy for sequencing. Second, the polyploid nature of wheat and the existence of homeologous sub-genomes sharing ~96%–98% identities in tetraploid or hexaploid wheat easily confound SNP detection, due to the existence of polymorphisms between them, known as “inter-homeologue polymorphisms” (IHP). Here, two restriction endonucleases (*MspI* and *PstI*) were used to generate digested fragments whose ligation to universal adapters, including “Y-shaped” adapters for the more common *MspI* overhang (due to the presence of *MspI*-*MspI* fragments), allowed for the specific amplification of *PstI*-*MspI* digested DNA fragments. The presence of a short 4-9bp barcode on the *PstI* adapter enabled multiplexed sequencing of the amplified DNA fragments on the Illumina sequencers. A total of 82 double haploid (DH) lines from the OWB mapping population in barley and 164 DH lines from the SynPoDH mapping population in wheat (a cross between the cultivar Opata85 and the hexaploid W9784 line) were sequenced, along with their respective parental lines. Bi-allelic SNPs were detected in both populations and a Fisher’s exact test of independence enabled the detection of putatively paralogous SNPs, as they are expected to segregate independently. The resulting bi-allelic SNPs then were added to the reference maps and placed on recombination bins if the parental information for the SNP of interest matched that of the bin markers for all lines present in that interval. A total of 34,396 bi-allelic SNPs, along with 241,159 sequence tags (treated as dominant markers) were added to the OWB map. In wheat, AntMap [134] was used to first create a GBS linkage map, where 1,485 SNP markers were assembled into 21 linkage groups representing the 21 wheat chromosomes. A total of 19,720 SNP markers and 367,423 sequence tags then were mapped on this newly created GBS map.

In another study, Harper *et al.* [135] developed a new concept, labeled as “associative transcriptomics”, in the complex polyploid genome of rapeseed (*Brassica napus*) where they used transcriptome sequencing (mRNA-Seq) for association studies. First, a pre-existing *B. napus* SNP linkage map [Bancroft] was used to improve the order and orientation of genome sequence scaffolds of diploid ancestors *B. rapa* (which contributed to the *B. napus* A genome) and *B. oleracea* (which contributed to the *B. napus* C genome), creating pseudomolecules representative of the polyploid *B. napus* chromosomes. The pseudomolecules were then used to infer gene order for a set of reference unigenes assembled *de novo* from a previous *B. napus* mRNA-Seq dataset [80]. *B. napus* mRNA-Seq data generated from an 84-line diversity panel were subsequently aligned to the reference unigenes, leading to the detection of 101,644 SNPs within 11,743 unigenes, out of which 62,980 were kept for further analysis, following the removal of SNPs with minor allele frequencies of less than 5%. These data were used in conjunction with the putative gene order on the *B. napus* pseudomolecules to study the genetic basis of two traits of interest (erucic acid content of seed oil and seed glucosinolate content). Diversity analysis on 53 of the *B. napus* accessions showed strong associations over previously identified QTLs for both traits. In addition, mRNA-Seq data also can be used to profile transcript abundance (thus enabling association studies with gene expression markers, or GEMs), and profiling data for the A and C genome copies of each unigene were used to detect unigenes (in one or both genomes) showing significant association between transcript abundance and glucosinolate content of seeds. Positioning SNP and GEM markers on the pseudomolecules identified two QTL regions containing orthologs of a transcription factor, known to control aliphatic glucosinolate biosynthesis in *A. thaliana*, whose loss by deletion causes a reduced seed glucosinolate phenotype in selected *B. napus* accessions.

In a separate study, Maughan *et al.* [136] re-sequenced two *Arabidopsis thaliana* parents and 58 RILs on the Roche 454 and Illumina platforms. Prior to sequencing, the genomic DNA from each individual first was digested with the restriction endonucleases *EcoRI* and *BfaI*, and the resulting DNA fragments were ligated to specific barcoded adapters, PCR amplified, pooled and size-selected. A total of 6,159 SNPs and 701 SNPs were discovered from the Roche 454 and Illumina data sets, respectively. 1,712 Roche 454 SNPs (selected after applying a 20% threshold for maximum missing data) were used for linkage mapping analysis. After removing a subset of SNPs showing either significant segregation distortion or linkage disequilibrium (LD) with other SNPs, pairwise linkage analysis grouped the remaining 1,555 SNPs into five distinct linkage groups (corresponding to all 5 chromosomes). The linkage order of the SNPs on the genetic map also was shown to be highly related to the order of the SNPs on the physical map.

In addition to resequencing segregating populations, GBS also has been used to sequence pools of mutants in bulked segregant analysis studies. In Arabidopsis, Schneeberger *et al.* [137] sequenced, via whole genome shotgun sequencing on the Illumina platform, a pool of 500 F_2 _plants generated by crossing a recessive ethane methyl sulfonate (EMS)-induced Col-0 mutant characterized by slow growth and light green leaves, with a wild type L*er* (Landsberg *erecta*) line. Interval analysis of the relative parental allele frequencies using the newly developed software package SHOREmap [138] revealed a narrow candidate region on chromosome 4. A mutation leading to a non-synonymous codon change in a putative gene of interest distant by only 4Kbp from the peak then was suggested as being the causal mutation for this particular phenotype. In a similar study, Austin *et al.* [139] used an Illumina whole genome shotgun sequencing approach to resequence three pools of 80 F_2_ cell wall-related mutants generated by crossing individuals corresponding to three separate EMS-induced Col-0 lines with a wild type L*er* mapping line. At least 230,000 SNPS were discovered for each mutant pool by aligning to the *A. thaliana* Col-0 reference genome. Regions of the genome lacking SNPs were discovered, corresponding to non-recombinant haplotypic blocks linked to the recessive mutations. A modification of the Illumina “chastity” statistic, which is normally used by the basecalling software to measure cross-talk between dyes during the sequencing process (and thus the “purity” of a specific base call at a given sequencing cycle) was then used to measure the proportion of reads that are completely homozygous for bases that differ from the reference genome, thus further narrowing the search window for a putative causative SNP of interest within these blocks. Finally, density interval analysis measuring the frequency of SNPs with discordant “chastity” values returned several non-synonymous SNPs for all three mutants, including three likely candidates located in putative genes of interest with roles ranging from actin cytoskeleton organization to sugar transport. Finally, Trick *et al.* [99] used a reduced-representation sequencing approach in wheat to identify SNPs between two parental lines in wheat and examine their frequency in two bulks of 28 homozygous recombinant lines differentiated by high and low grain protein contents, respectively. SNP detection first was performed by re-sequencing with the Illumina sequencing technology the transcriptome (“mRNA-Seq”) of two parental lines, LDN and RSL65, segregating for the trait of interest. Individual sequences from each parental then were mapped to the NCBI wheat unigene dataset separately, thus creating two SNP sets, and a custom Perl script was used to determine differences between the two parental SNP sets. IHPs representing a consensus sequence with the same ambiguity code in each parental line and common between the two parental sets were removed, leading to the identification of 3,963 putative SNPs between LDN and RSL65. This dataset was later reduced to 3,427 SNPs, after examining SNP frequency distribution for each mapped unigene, and the possibility of mapping putative SNPs to closely related paralogues. mRNA-seq data then were generated for each bulk (“high” and “low” protein contents) in order to compare the allelic frequencies for the parental SNPs between the two of them. Relative SNP frequency measurements led to the characterization of two tightly linked unigenes located on wheat chromosome arm 6BS.

## 8. Conclusions

High-throughput variant discovery has been made possible in multiple species by the recent advent of next-generation DNA sequencing technologies. Continuous increase in sequencing throughput and the accompanying decrease in consumable cost per Gbp has allowed researchers to switch focus from resequencing small panels of parental individuals for the sole purpose of discovering variants to resequencing much larger pools of individuals within a population, where the sequenced differences are used directly as genotypic markers. This genotyping-by-sequencing (GBS) approach has several advantages, including the facts that no preliminary sequence information is required and that all newly discovered markers originate from the population being genotyped. On the other hand, due to several biological and technical factors, such as PCR amplification bias during the library construction step, not all sequenced regions of interest are evenly covered in all individuals within a population, reaffirming the need for imputing missing data using pedigree or parental information when available.

Because DNA fragments are more readily prepared using a genome-wide approach (as opposed to a targeted approach where only a small region of the genome is sequenced), the advent of GBS is expected to have a more profound impact on mapping strategies benefiting from a dense genome-wide distribution of markers. Such strategies include Genome-Wide Association Studies (GWAS), Bulked Segregant Analysis (BSA) and Genomic Selection (GS). 

Successive improvements of the sequencing chemistries and basecalling software are allowing NGS technologies to deliver higher sequencing throughputs per run, which in turn enables deeper multiplexing for a fixed average sequencing depth per sample. Although the cost of sample preparation and bioinformatics analysis are not decreasing as rapidly as the cost of sequencing, such a trend is already enabling GBS to be a cost-competitive alternative to other whole-genome genotyping platforms. It is expected that, as the amount and quality of sequencing information generated per run keeps increasing, thus allowing even higher plexing and lower costs per samples, plant breeders soon may be able to sequence even larger populations, allowing genomic selection or the determination of a population structure without prior knowledge of the diversity present in the species [93]. 
